# Development and Mechanism of the Graded Polymer Profile-Control Agent for Heterogeneous Heavy Oil Reservoirs Under Water Flooding

**DOI:** 10.3390/gels11110856

**Published:** 2025-10-26

**Authors:** Tiantian Yu, Wangang Zheng, Xueqian Guan, Aifen Li, Dechun Chen, Wei Chu, Xin Xia

**Affiliations:** 1 School of Petroleum Engineering, China University of Petroleum (East China), Qingdao 266580, China; yutiantian.slyt@sinopec.com (T.Y.); chendc@upc.edu.cn (D.C.); 2 Shandong Provincial Key Laboratory of Heavy Oil Production Technology, Dongying 257000, China; zwg0124@126.com (W.Z.); guanxueqian.slyt@sinopec.com (X.G.); chuwei333@163.com (W.C.); xiaxin27.slyt@sinopec.com (X.X.); 3 Petroleum Engineering Technology Research Institute of Shengli Oilfield Branch, Dongying 257000, China

**Keywords:** profile-control agent, emulsion blocking, graded profile control, heavy oil, hydrophobic association

## Abstract

During water flooding processes, the high viscosity of heavy oil and significant reservoir heterogeneity often lead to severe water channeling and low sweep efficiency. Addressing the limitations of traditional hydrophobically associating polymer-based profile-control agents—such as significant adsorption loss, mechanical degradation during reservoir migration, resulting in a limited effective radius and short functional duration—this study developed a polymeric graded profile-control agent suitable for highly heterogeneous conditions. The physicochemical properties of the system were comprehensively evaluated through systematic testing of its apparent viscosity, salt tolerance, and anti-aging performance. The microscopic oil displacement mechanisms in porous media were elucidated by combining CT scanning and microfluidic visual displacement experiments. Experimental results indicate that the agent exhibits significant hydrophobic association behavior, with a critical association concentration of 1370 mg·L^−1^, and demonstrates a “low viscosity at low temperature, high viscosity at high temperature” rheological characteristic. At a concentration of 3000 mg·L^−1^, the apparent viscosity of the solution is 348 mPa·s at 30 °C, rising significantly to 1221 mPa·s at 70 °C. It possesses a salinity tolerance of up to 50,000 mg·L^−1^, and a viscosity retention rate of 95.4% after 90 days of high-temperature aging, indicating good injectivity, reservoir compatibility, and thermal stability. Furthermore, within a concentration range of 500–3000 mg·L^−1^, the agent can effectively emulsify Gudao heavy oil, forming O/W emulsion droplets with sizes ranging from 40 to 80 μm, enabling effective plugging of pore throats of corresponding sizes. CT scanning and microfluidic displacement experiments further reveal that the agent possesses a graded control function: in the near-wellbore high-concentration zone, it primarily relies on its aqueous phase viscosity-increasing capability to control the mobility ratio; upon entering the deep reservoir low-concentration zone, it utilizes “emulsion plugging” to achieve fluid diversion, thereby expanding the sweep volume and extending the effective treatment period. This research outcome provides a new technical pathway for the efficient development of highly heterogeneous heavy oil reservoirs.

## 1. Introduction

In heavy oil recovery, water flooding technology is widely applied due to its low cost and ease of implementation. However, the high viscosity of heavy oil and the unfavorable water-oil mobility ratio easily lead to channeling and fingering, resulting in low sweep efficiency and severely restricting the efficient development of heavy oil resources. To improve development outcomes, injecting efficient profile-control agents has become a key technological means for enhancing oil recovery [1]. Currently used agents primarily fall into two categories: one is polymer-based agents [2,3,4], which improve the water-oil mobility ratio by increasing aqueous phase viscosity and are widely used in many conventional heavy oil reservoirs; the other includes gel, microsphere, and particulate agents [5,6,7,8], which rely on physical blockage to inhibit fingering and enlarge the sweep volume, making them more suitable for high-water-cut reservoirs [9].

Polymer-based agents dominate in conventional water-flooded heavy oil reservoirs due to their strong adaptability and flexible control. For hydrophobically associating polymers based on acrylamide, when their concentration exceeds the critical association concentration (CAC), intermolecular association occurs, forming a “dynamic physical cross-linked network structure” that exhibits excellent viscosity-increasing and shear-resistant properties. For example, the sulfonic acid group-containing hydrophobically associating polymer developed by Gou Shaohua et al. [10] retained over 80% viscosity under high temperature (180 °C) and high salinity (18 × 10^4^ mg/L), with a viscosity-increasing effect 3–5 times that of conventional polyacrylamide; the copolymer containing vinyl-fused aromatic ring monomers developed by Zhong Chuanrong et al. [11] remained stable at 150 °C and 20 × 10^4^ mg/L salinity, with a moderate molecular weight suitable for the pore-throat conditions of medium-low permeability reservoirs. Zhu Linyong et al. [12] synthesized an amphiphilic polyether dendron/polyacrylic acid block copolymer whose hydrophobic groups associate in water, significantly increasing solution viscosity; Fu Meilong et al. [13] prepared a hydrophobically associating polymer using cationic hydrophobic monomers and systematically studied its microscopic aggregation state and rheological properties. However, such agents have a single function, primarily relying on viscosity increase, are prone to viscosity loss due to shear in the formation, have a limited radius of action, and a short effective period for profile control and displacement [14,15].

Based on research, Qu Pingping [16] developed a highly adaptable asphalt emulsion profile control system, utilizing the adjustable particle size of emulsified asphalt particles to achieve profile control functionality; Zhang Wei [17] explored a novel enhanced dispersion system profile control technology, verifying its ability to improve sweep volume; Li Yan [18] focused on the long-distance transport characteristics of an online profile control composite emulsion, highlighting the importance of interfacial stability and fluidity for deep profile control.

The betaine-hydrophobically associating monomer copolymer system developed by Yang Hongbin et al. [19] utilizes the “anti-polyelectrolyte effect” to form a stable supramolecular structure under high salinity conditions, achieving an aqueous phase viscosity of 61.2 mPa·s and a viscosity reduction rate exceeding 80%. This system focuses on constructing small-sized emulsions to enhance viscosity reduction and transport capability, but the small particle size hinders effective blockage of large channels, limiting sweep volume expansion and overall profile control effectiveness. Furthermore, existing evaluations mostly rely on macroscopic core experiments [20,21], lacking microscopic characterization methods, which restricts mechanism research and performance optimization.

Addressing the bottlenecks in existing profile control technologies, this study synthesizes a novel polymer system with multi-stage control functionality. This system significantly increases aqueous phase viscosity at high concentrations to improve the mobility ratio; at low concentrations, it can form large-sized emulsions with heavy oil, enlarging the sweep volume through plugging effects and extending the effective period, achieving a graded profile control strategy combining “aqueous phase viscosity increase” and “emulsion plugging”. This paper first evaluates the solution properties of the profile control agent under different temperatures and salinities, determining its CAC as 1370 mg·L^−1^, and systematically investigates its emulsification behavior and emulsion stability. Furthermore, combining CT scanning and microfluidic displacement experiments, it reveals the microscopic mechanisms of emulsion plugging and fluid diversion, providing a technically feasible and mechanistically clear new approach for efficient heavy oil development.

## 2. Results and Discussion

### 2.1. Solution Properties

**Figure 1:**
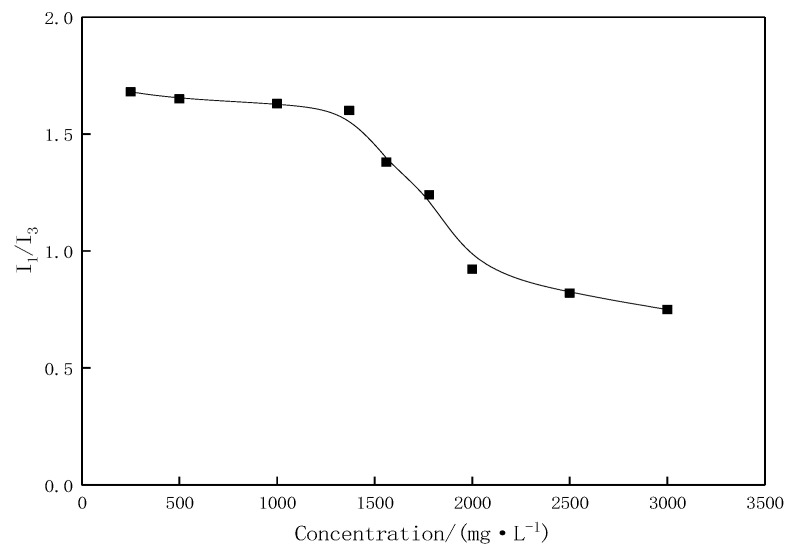
Curve of I_1_/I_3_ change with concentration of profile control agent.

The critical association concentration (CAC) of profile-control agent was determined by pyrene fluorescence probe method. As shown in Figure 1, the I_1_/I_3_ ratio of pyrene showed a significant turning point when the polymer concentration reached 1370 mg·L^−1^. When the concentration was lower than this value, I_1_/I_3_ decreased slowly, indicating that there was a small amount of intramolecular association; When the concentration is higher than this value, I_1_/I_3_ decreases sharply, indicating the formation of a large number of aggregates dominated by intermolecular hydrophobic association. Therefore, the CAC of the profile control agent was determined to be 1370 mg·L^−1^.

**Figure 2:**
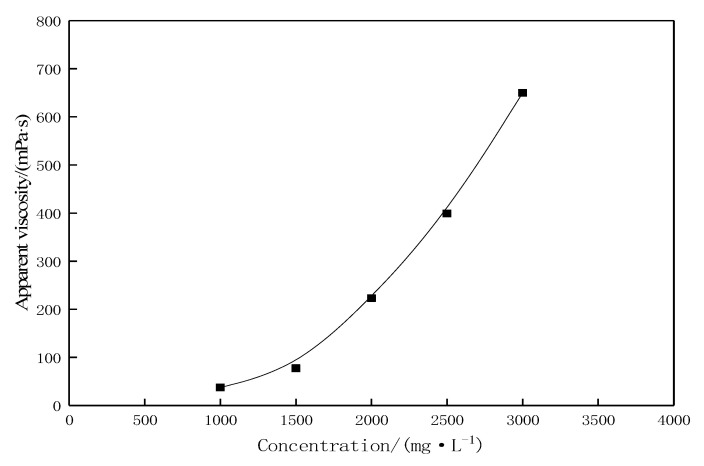
Curve of apparent viscosity change with concentration of profile control agent at 50 °C.

**Figure 3:**
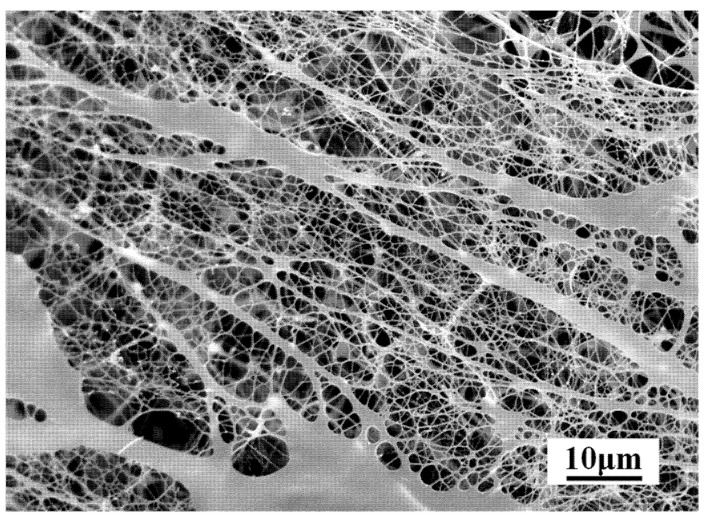
SEM image of the profile-control agent solution at a concentration of 3000 mg·L^−1^.

At a fixed shear rate of 7.34 s^−1^, the apparent viscosity of the novel profile-control agent solution at different concentrations is shown in Figure 2. As the concentration of the agent increases, the apparent viscosity of the solution gradually rises. The high viscosity characteristics primarily stem from the following three reasons: First, the large molecular volume of the agent itself hinders the free movement of water molecules. Second, the macromolecules of the agent undergo solvation, trapping a significant amount of “free” liquid. The molecular chains adopt a regularly loose coil-like structure in the solution, with a large number of water molecules contained inside the coils and the formation of a thick hydration layer, leading to a significant increase in hydrodynamic volume and higher flow resistance [22]. Third, interactions exist between the macromolecules. When the concentration of the agent exceeds the CAC, the hydrophobic groups AS on the side chains of the molecular chains undergo intermolecular association due to strong interactions, forming a certain pseudo-network structure, thereby increasing the flow resistance of the solution and causing a sharp rise in apparent viscosity. SEM results (Figure 3) reveal the formation of a hydrophobic association-induced three-dimensional network structure in the solution at this stage. This associative behavior is key to achieving effective profile control and displacement performance.

**Figure 4:**
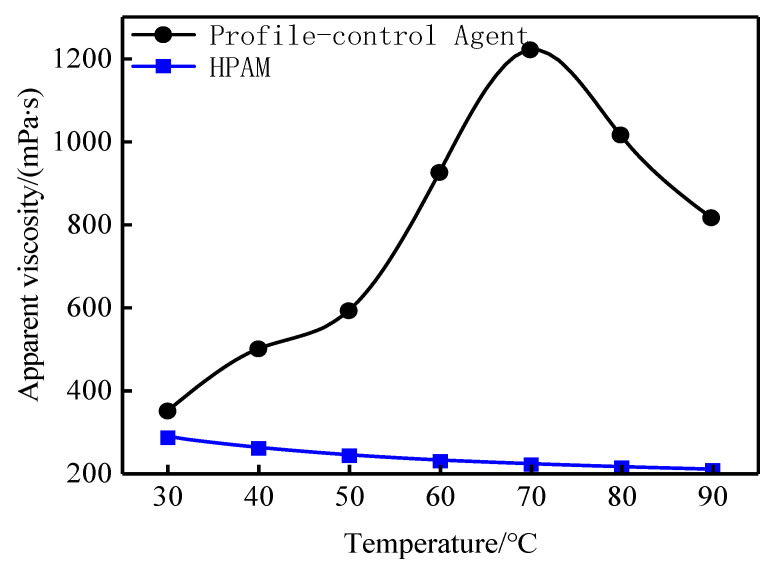
Variation in viscosity with temperature for profile-control agent at 3000 mg·L^−1^.

Figure 4 shows the effect of temperature on the apparent viscosity of the profile-control agent solution at a fixed concentration of 3000 mg·L^−1^. The apparent viscosity was 348 mPa·s at 30 °C and increased significantly to 1221 mPa·s at 70 °C. The low viscosity at 30 °C ensures good injectivity, while the substantial viscosity increase within the typical reservoir temperature range of 50–80 °C effectively improves the oil-water mobility ratio. This “low viscosity at low temperature, high viscosity at high temperature” rheological behavior is opposite to the thermal thinning exhibited by commonly used partially hydrolyzed polyacrylamide (HPAM) [23]. This distinct thermothickening behavior is attributed to the agent’s specific molecular structure, which contains hydrophobic (AMC_16_S), anionic sulfonate, and carboxylate groups. The association of the hydrophobic groups is an endothermic process; thus, heating promotes intermolecular association and leads to the formation of microgel (a positive effect). Concurrently, elevated temperature intensifies the motion of ionic groups, enhancing electrostatic repulsion and expanding the molecular chains—another positive effect. Opposing these are negative effects: enhanced thermal motion of hydrophobic groups weakens their association, while increased water molecule motion reduces hydrophilic group hydration, causing chain contraction. At elevated temperatures, the solution’s viscosity peaks when the positive and negative effects balance. A further temperature increase tilts this balance, weakening the association and ultimately reducing viscosity.

**Figure 5:**
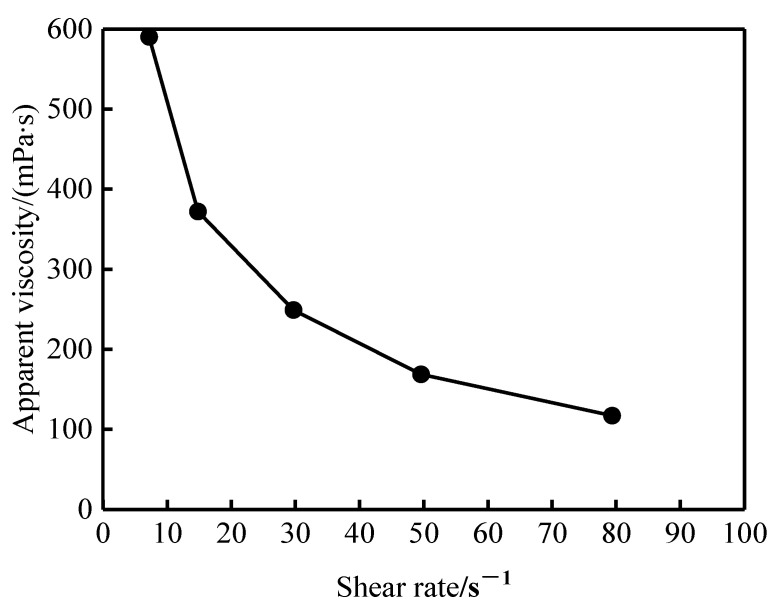
Apparent viscosity curves of profile-control agent under different shear rate conditions.

Figure 5 illustrates the shear-thinning behavior of the profile-control agent solution. The apparent viscosity decreased significantly with increasing shear rate. Specifically, at 50 °C, the viscosity measured 590 mPa·s at 7.34 s^−1^ but fell to 113 mPa·s at 80 s^−1^. This phenomenon originates from the reversible dissociation of the dynamic network structure formed by molecular association under high shear, resulting in reduced flow resistance. It is noteworthy that even under strong shear, the solution maintains an effective viscosity of 113 mPa·s, indicating excellent shear stability. This property ensures the structural integrity of the profile-control agent during wellbore perforation and reservoir percolation processes, thereby providing reliable profile control performance under actual reservoir conditions.

### 2.2. Salt Resistance

**Figure 6:**
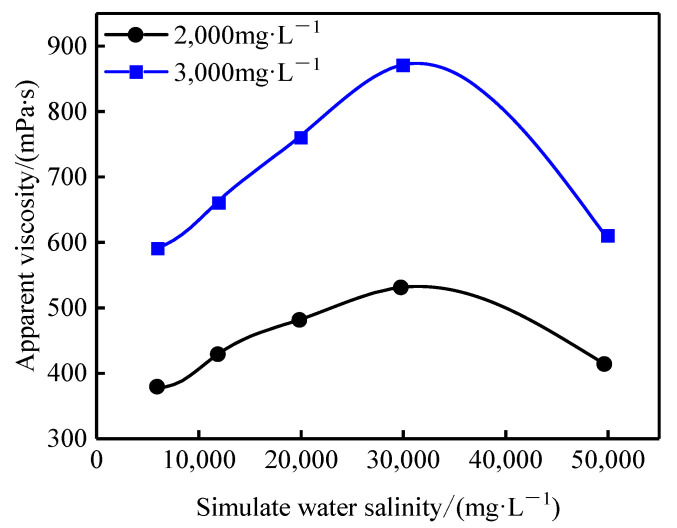
Apparent viscosity curves of profile-control agent under different salinity conditions.

Simulated brine with different salinities was prepared using NaCl, CaCl_2_, and MgCl_2_ to investigate the salt tolerance of the profile-control agent. As shown in Figure 6, the apparent viscosity of the agent solutions at both concentrations exhibited a non-monotonic trend, initially increasing and then decreasing with rising salinity. For the 3000 mg·L^−1^ solution, the apparent viscosity increased from 590 mPa·s at 6000 mg·L^−1^ salinity to a maximum of 870 mPa·s at 30,000 mg·L^−1^ salinity, before decreasing to 610 mPa·s at 50,000 mg·L^−1^ salinity.

This behavior is governed by the dual role of salt ions in regulating polymer molecular conformation and aggregation state [24]. At moderate salinities, cations shield the negative charges on the carboxylate and sulfonate groups, reducing electrostatic repulsion between chains. This causes the molecular chains to coil, promoting intermolecular association of hydrophobic groups and the formation of a dense network structure, thereby significantly increasing viscosity [25]. In contrast, at excessively high salinities, an over-abundance of ions disrupts the hydration layer around the chains and compresses the electric double layer. This leads to the dissociation of hydrophobic microdomains and excessive chain coiling, resulting in a reduced hydrodynamic volume and a consequent decline in viscosity.

**Figure 7:**
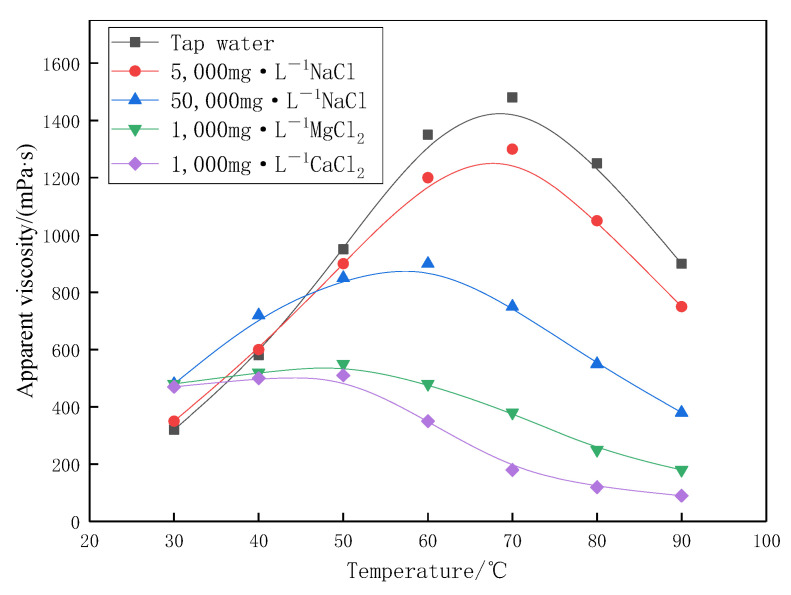
Apparent viscosity curves of the profile-control agent under different ionic compositions.

The differential effects of Na^+^, Ca^2+^, Mg^2+^ on the viscosity of profile-control agent solution are shown in Figure 7. Under the condition of 30–90 °C, the viscosity peak of tap water system and 5000 mg·L^−1^ NaCl system is relatively high. Under the condition of low salt, the synergistic effect of electrostatic repulsion and hydrophobic association makes the viscosity peak at 70 °C (>1300 mPa·s), which is consistent with the experimental results in Figure 5 above. In contrast, the viscosity peak value of the 50,000 mg·L^−1^ NaCl system decreases, and the corresponding temperature value decreases to 60 °C. The concentration of NaCl promotes the molecular chain curl by shielding electrostatic repulsion, shortens the spacing of hydrophobic groups, resulting in the association of hydrophobic groups at a lower thermal motion temperature (60 °C), which weakens and advances the peak value. Compared with NaCl system, the peak viscosity of 1000 mg·L^−1^ CaCl_2_ and MgCl_2_ system profile-control agents in Figure 7 is further reduced, and the corresponding temperature value is further reduced to 50 °C. At the molecular level, the influence of Ca^2+^ and Mg^2+^ divalent cations on the microgel network is essentially different from that of Na^+^. Divalent ions not only have electrostatic shielding effect but also have specific strong interaction with the negatively charged carboxyl groups on the polymer chain. Especially, Ca^2+^ can act as an ion bridge to induce the formation of local, too strong cross-linking points between chains. On the one hand, this “ion bridge” effect will fix the molecular chain conformation, inhibit the dynamic reversible association and dissociation of hydrophobic groups, and greatly weaken the hydrophobic association. On the other hand, under the thermal or shear disturbance, the microgel structure is easy to be destroyed from these positions, resulting in a sharp decline in viscosity, further reducing the peak and advancing to about 50 °C.

### 2.3. Thermal Stability

At a temperature of 65 °C, the apparent viscosity and retention rate of the 3000 mg·L^−1^ profile-control agent solution under different aging times are shown in Table 1. After being placed at a constant temperature (65 °C) for 90 days under anaerobic conditions, the solution maintained an apparent viscosity of 1073 mPa·s, corresponding to a high viscosity retention rate of 95.4%. This result demonstrates long-term thermal stability far superior to that of HPAM, a performance advantage originating from a fundamentally different viscosity-building mechanism [26].

The viscosity of HPAM relies primarily on the chemical integrity of its molecular chains, which are susceptible to irreversible hydrolytic degradation at high temperatures. In contrast, when the concentration of this profile-control agent exceeds its critical association concentration (CAC, 1370 mg·L^−1^), it forms a dynamic and reversible physical cross-linking network via hydrophobic associations. Although elevated temperature can weaken some associations, the hydrophobic groups continuously re-associate, granting the network self-healing capabilities. This mechanism effectively circumvents the chemical degradation issue inherent to HPAM, enabling long-term viscosity stability and sustained profile control performance in high-temperature reservoir environments [27].

### 2.4. Adsorptivity

**Figure 8:**
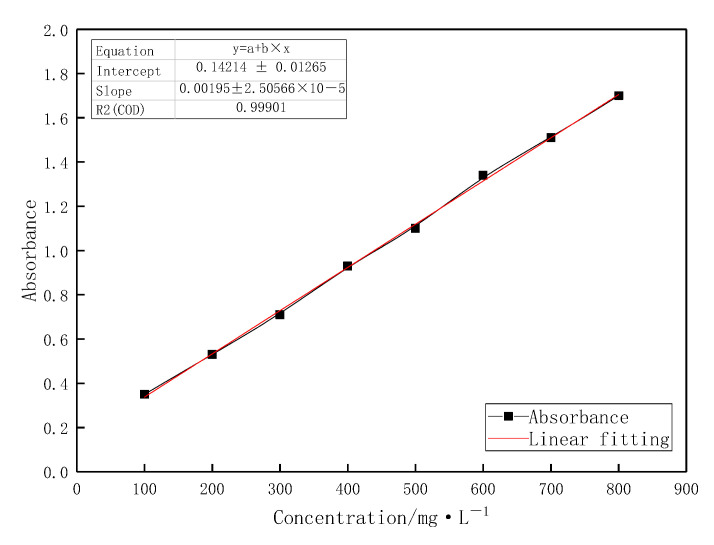
Standard curve of the relationship between the concentration of the profile-control agent and absorbance.

**Figure 9:**
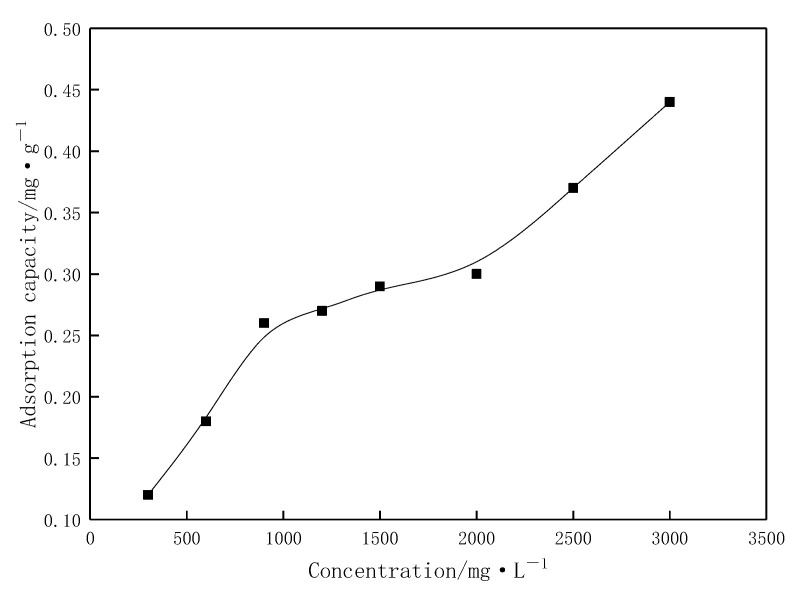
Static adsorption amount of the profile-control agent on the quartz sand surface at different concentrations.

A standard curve correlating the concentration of the profile-control agent with absorbance was established using the starch-chromium iodide colorimetric method, and the static adsorption amount of the agent on quartz sand surfaces was determined at different concentrations. As shown in Figure 8, the mass concentration of the agent exhibits a strong linear relationship with absorbance, with an R^2^ value exceeding 0.99. Figure 9 demonstrates that the adsorption amount on the quartz sand surface increases gradually with rising agent concentration. The agent molecules undergo monolayer adsorption on the solid surface, reaching the first adsorption equilibrium at a concentration of 9000 mg/L, which aligns with the Langmuir adsorption isotherm model [28,29]. When the concentration is further increased beyond 15,000 mg/L, the adsorption amount continues to rise. In this regime, agent molecules in the solution can be indirectly adsorbed at the solid–liquid interface by associating intermolecularly with agent molecules already adsorbed at the interface. This manifests as multilayer adsorption of the agent molecules at the interface, leading to a further increase in adsorption amount. During transport through porous media in reservoirs, the profile-control agent undergoes depletion due to adsorption, resulting in a reduction in its effective concentration. This, in turn, affects the viscosity of the solution and subsequent emulsification behavior.

### 2.5. Emulsification Behavior

**Figure 10:**
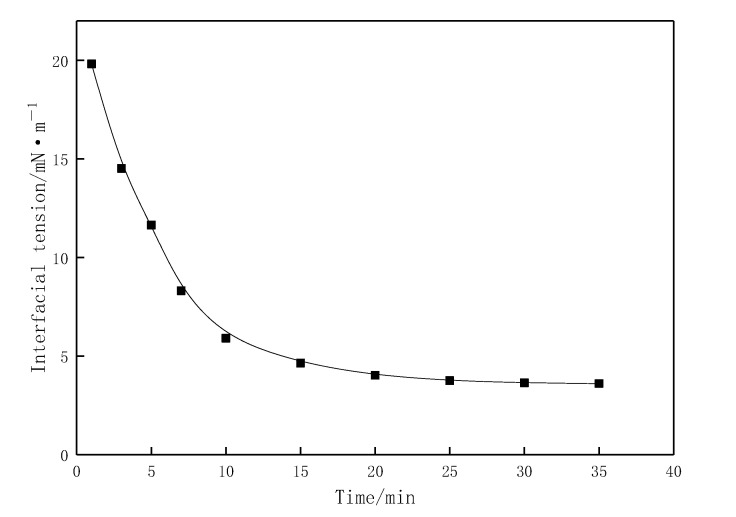
Interfacial tension between the aqueous solution of the profile-control agent and crude oil as a function of time.

The interfacial tension curve between the aqueous solution of the profile-control agent and crude oil is shown in Figure 10. The equilibrium interfacial tension of the agent is 3.65 mN/m, indicating a certain level of interfacial activity, which forms the basis for emulsifying crude oil.

**Figure 11:**
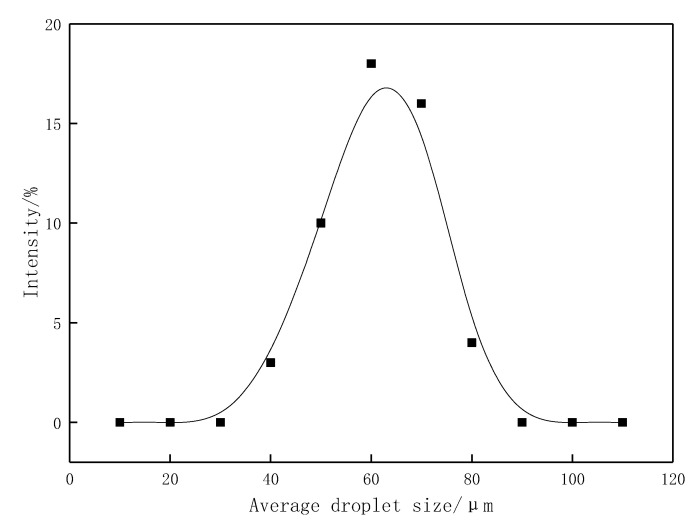
Droplet size distribution of crude oil emulsions.

**Figure 12:**
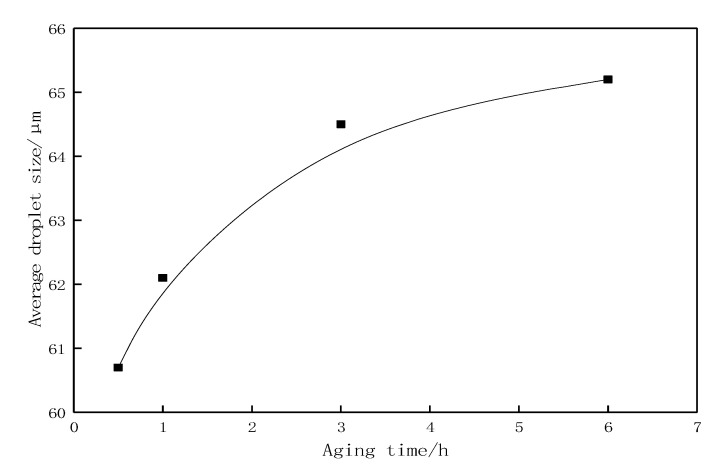
Variation curve of emulsion droplet size with aging time.

With a fixed oil-to-water ratio of 7:3, a 1000 mg·L^−1^ solution of the profile-control agent mixed with Gudao crude oil was able to form a low-viscosity O/W emulsion, with droplet sizes distributed in the range of 40–80 μm (Figure 11). Considering the practical requirements for oilfield applications, the crude oil emulsion must possess dynamic shear stability under reservoir shear conditions to ensure an effective duration of action. The variation in emulsion droplet size with aging time at 50 °C and a constant shear rate of 7.34 s^−1^ is shown in Figure 12. As aging time increased, the droplet size gradually increased and essentially stabilized after 3 h of aging. When the aging time increased from 0.5 h to 6 h, the droplet size increased from 60.7 μm to 65.2 μm, representing a 7.4% increase, which indicates that the emulsion possesses a certain degree of dynamic stability.Furthermore, regulations in oilfields such as Shengli stipulate that chemical agents present in produced fluid must not interfere with the surface treatment system. Under settling conditions (static), the emulsion must exhibit efficient demulsification capability. According to the standard (Experimental methods refer to appendix standard references [30]), the crude oil emulsion formed by this profile-control agent achieved a dehydration rate of 81.5% after 1 h of static settling.

**Figure 13:**
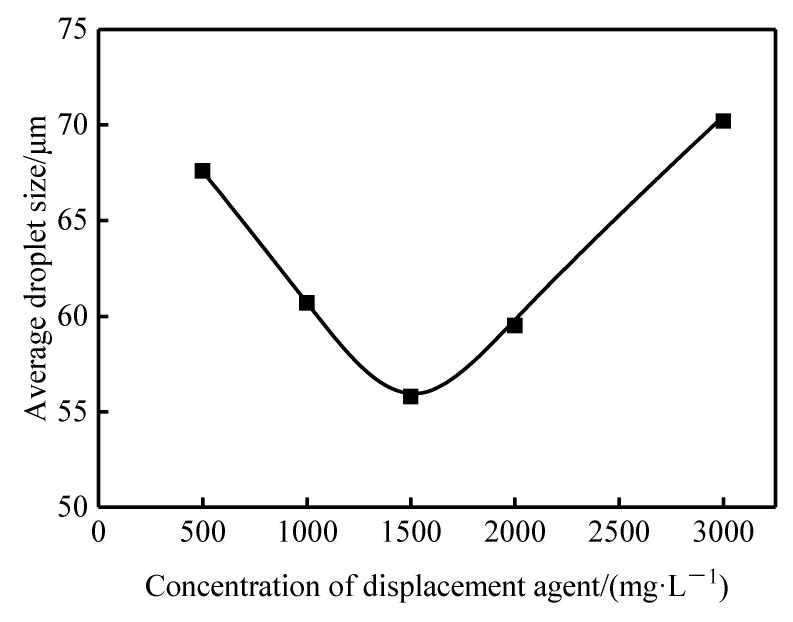
Average droplet size of emulsion droplets formed at different concentrations.

The influence of the profile-control agent concentration on the average droplet size of the crude oil emulsion is shown in Figure 13. When the agent concentration increased from 500 mg·L^−1^ to 1500 mg·L^−1^, the average droplet size of the emulsion decreased significantly from 67.6 μm to 55.8 μm. This reduction stems from the enhanced interfacial film effect resulting from the increased density of adsorbed molecules at the interface [31]. However, when the concentration exceeded the critical value of 1500 mg·L^−1^, the average droplet size exhibited an anomalous increasing trend. At this point, the sharp rise in the bulk viscosity of the system causes kinetic factors to become dominant. The high viscosity severely restricts the diffusion rate of the agent molecules toward the oil-water interface and their orderly arrangement process at the interface, thereby reducing emulsification efficiency [32]. Consequently, within the deep reservoir, where the agent concentration is significantly reduced due to losses such as rock adsorption and dilution, emulsifying crude oil becomes considerably less difficult, facilitating the formation of an O/W emulsion.

### 2.6. Blocking Capacity

**Figure 14:**
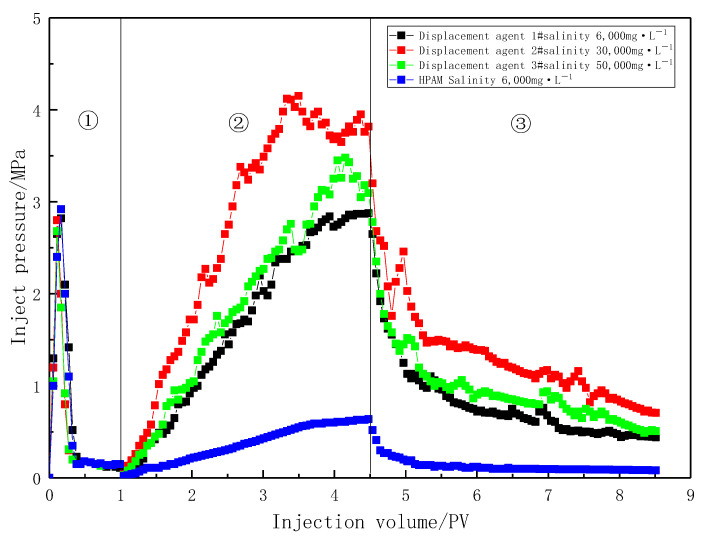
Variation curve of injection pressure with injection volume at different development stages. ① water flooding; ② agent flooding; ③ Subsequent water flooding.

To evaluate the blocking capacity of the profile-control agent, core flooding experiments were conducted using a 1500 mg·L^−1^ solution under different salinity conditions, and the results were compared with those from HPAM flooding. For a consistent comparison, the HPAM concentration was set at 2400 mg/L, as the viscosity of the HPAM solution at this concentration is comparable to that of Agent #1. The experimental results are shown in Figure 14.

Among the three agent solutions, at the end of agent flooding (4.5 PV), the injection pressure for Agent #2 was the highest, while that for Agent #1 was the lowest. This indicates that as salinity increased from 6000 mg·L^−1^ to 50,000 mg·L^−1^, the blocking strength of the agents first increased and then decreased. This trend is consistent with the observations in Figure 7, where the highest blocking strength occurred at a salinity of 30,000 mg·L^−1^, corresponding to the peak solution viscosity. Solution viscosity is the fundamental factor determining flow resistance.

Furthermore, at the end of agent flooding (4.5 PV), the injection pressure of the profile-control agent solution was 3.5 to 5 times higher than that of HPAM with an equivalent apparent viscosity. Even after subsequent water flooding (8.5 PV), the displacement pressure remained higher than that of HPAM. Additionally, emulsions were observed in the effluent during agent flooding, whereas no such emulsions were detected in the HPAM effluent. This demonstrates that while the agents increase displacement pressure through their bulk viscosity, they also achieve effective blocking via in situ emulsification, where emulsion droplets plug pores.

It is worth noting that the displacement pressure curve for HPAM was smooth and stable, characteristic of typical viscous fluid behavior. In contrast, the pressure curves for the three agents showed significant and frequent fluctuations during both the rising and declining phases. These pressure fluctuations are a typical signature of the dynamic process where discrete, movable emulsion droplets undergo “plugging-breakthrough-re-plugging” at pore throats. The temporary trapping of a single droplet at a pore throat causes an instantaneous pressure rise, while its subsequent deformation, extrusion, or breakthrough under driving pressure leads to an instantaneous pressure drop. This phenomenon further confirms the significant contribution of emulsion plugging to the overall blocking effect.

### 2.7. Oil Displacement Mechanisms

**Figure 15:**
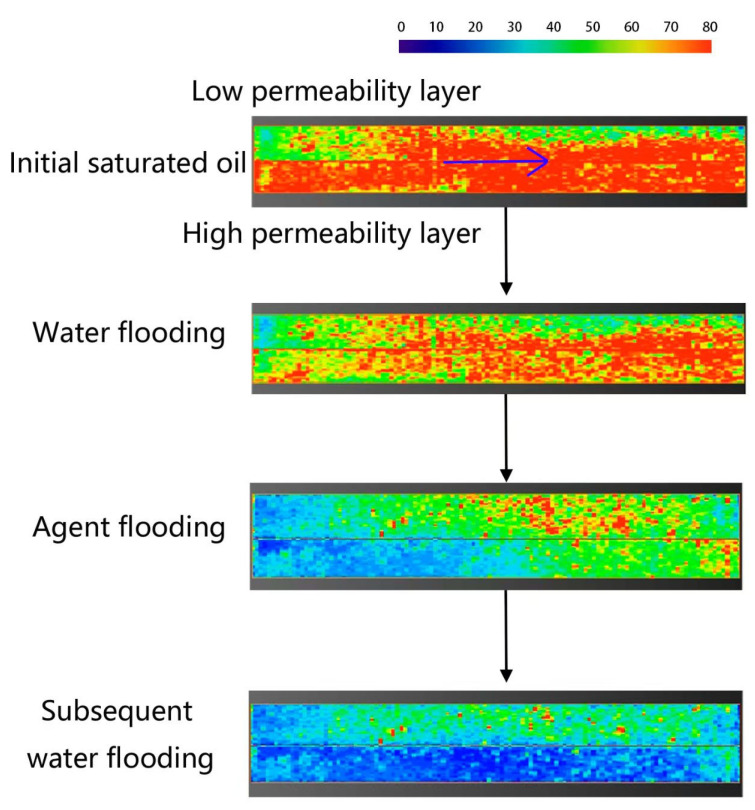
CT scan oil saturation images of high permeability and low permeability rock cores at different displacement stages. (The blue arrows indicate the direction of displacement.)

**Figure 16:**
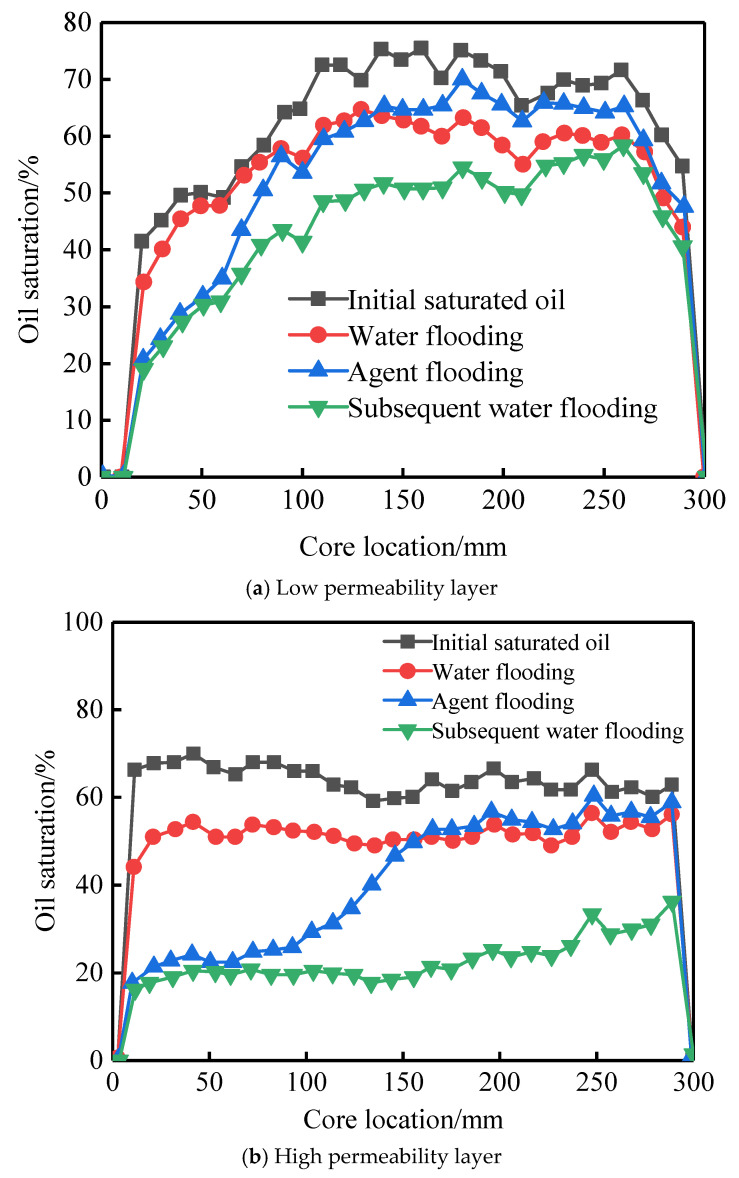
Oil saturation profiles of high- and low-permeability cores at different displacement stages.

Core CT scanning experiments were conducted to study the evolution of oil saturation in high- and low-permeability layers at different displacement stages. Based on the oil saturation data, the Dykstra-Parsons coefficient (Vdp), which characterizes the heterogeneity of spatial oil saturation distribution, was calculated. The calculation method of this index draws on the classical permeability Dykstra-Parsons coefficient and is applied here to the oil saturation field. The Vdp coefficient ranges from 0 to 1. A value of 0 indicates a very uniform oil saturation distribution in the reservoir, while a value of 1 indicates a very heterogeneous distribution. The CT scan results and Vdp coefficient calculations are shown in Figure 15 and Figure 16, and Table 2.

During the initial oil saturation stage, influenced by the pore structure, the low-permeability layer was more difficult to saturate, resulting in a Vdp coefficient of 0.2, higher than the 0.1 in the high-permeability layer. This indicates that the low-permeability layer had stronger inherent saturation heterogeneity in its initial state.

In the water flooding stage, due to the oil-water mobility ratio, injected water preferentially formed dominant flow channels in the high-permeability layer. The overall oil saturation decreased by only 6–15%. The Vdp coefficients for the high- and low-permeability layers increased to 0.22 and 0.36, respectively, indicating that the water flooding process further exacerbated intra-layer heterogeneity and significantly enhanced fingering.

During the profile-control agent flooding stage, the agent preferentially entered the high-permeability layer. CT images showed a significant decrease in oil saturation near the core inlet, with saturation gradually increasing with distance. Cross-over points between the water flooding and agent flooding profiles appeared at 132 mm in the low-permeability layer and 154 mm in the high-permeability layer, indicating that the agent effectively mobilized oil near the inlet and pushed it deeper, forming an oil saturation bank. In this stage, the Vdp coefficient for the high-permeability layer sharply increased to 0.75, suggesting highly uneven flow paths due to emulsion blockage, successfully achieving fluid diversion. The Vdp coefficient for the low-permeability layer was 0.57, also reflecting significant reorganization of its internal flow structure.

Dynamic analysis of the effluent from the high-permeability layer further revealed the staged evolution of the displacement mechanism. In the early displacement stage (0.3 PV), with low adsorption loss of the agent, the system viscosity was high, and the emulsion droplet size was large (86 μm). Under the synergistic effect of high viscosity and large droplets, the blocking pressure reached 434 kPa. By the middle and late displacement stages (0.5 PV), as agent adsorption loss increased, the system viscosity decreased, and the emulsion droplet size reduced to 64 μm. The blocking pressure correspondingly dropped to 407 kPa, indicating that blocking intensity is positively correlated with both droplet size and system viscosity.

In the subsequent water flooding stage, initially, because the displacement system still occupied part of the high-permeability channels, injected water bypassed, enlarging the sweep volume. As flooding progressed, the high-permeability layer, having lower flow resistance, broke through first, re-establishing dominant channels. Injected water channeled again, and oil mobilization in the low-permeability layer nearly stagnated. In this stage, the Vdp coefficients for the high- and low-permeability layers decreased to 0.38 and 0.41, respectively, indicating that part of the emulsion blockage was breached, and intra-layer heterogeneity was somewhat mitigated.

At the end of the experiment, the remaining oil saturation in most areas of the high-permeability layer dropped below 20%, while it remained at a relatively high level of 20–60% in the low-permeability layer. This fully demonstrates the controlling influence of inter-layer physical property differences on recovery efficiency.

### 2.8. Visualized Migration Characteristics

**Figure 17:**
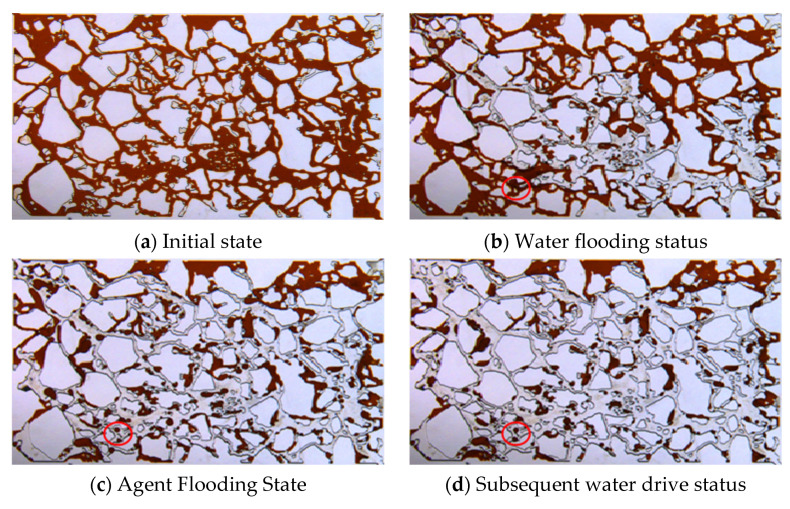
Microfluidic experimental results of 3000 mg·L^−1^ profile control agent.

**Figure 18:**
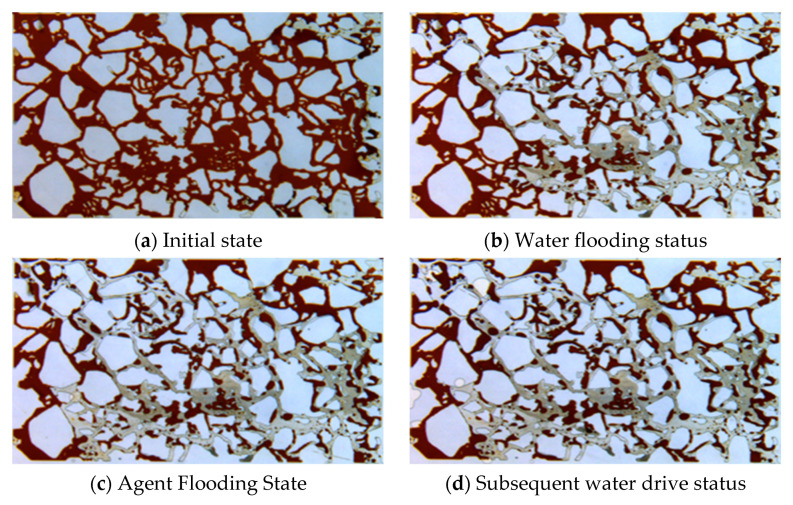
Microfluidic experimental results of 1500 mg·L^−1^ profile control agent.

Microfluidic experiments (Figure 17 and Figure 18) were used to visualize the oil displacement process governed by the profile-control agent. During the initial water flooding stage, the injected fluid preferentially channeled through high-permeability pathways, resulting in significant oil bypass and the formation of unswept zones on the flanks. Injection of the profile-control agent solutions at 3000 mg·L^−1^ and 1500 mg·L^−1^ substantially altered the flow dynamics. By leveraging the synergistic effects of aqueous-phase viscosity enhancement and in situ emulsion plugging, the displacement front was effectively diverted. This successfully mobilized the residual oil in the previously unswept areas and significantly improved the sweep efficiency. The agent at the higher concentration (3000 mg·L^−1^) achieved a superior displacement efficiency of 40.2%, which was 7.9% higher than that of the 1500 mg·L^−1^ system. This result underscores that the agent concentration is a critical factor for effectively accessing and mobilizing oil from smaller pores. A comparative analysis with HPAM flooding at an equivalent concentration (Table 3) highlighted the exceptional emulsification capability of the profile-control agent. The emulsions it generated were characterized by a large population of droplets (172), a broad size distribution, and a high relative content (28.79%). In contrast, HPAM flooding yielded negligible emulsification. The sequential images of the oil phase within the red-circled area in Figure 17 further elucidate the underlying “plugging” mechanism: the agent emulsifies the residual oil into discrete droplets. These droplets then migrate under the drive of subsequent water injection, eventually becoming lodged at pore throats. This plugging action diverts the flow field, thereby activating the surrounding residual oil.

### 2.9. Microscopic Oil Displacement Mechanism

**Figure 19:**
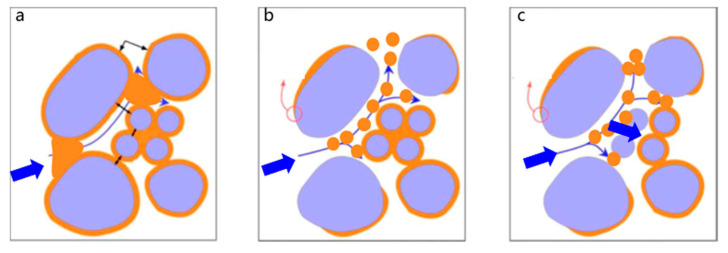
Emulsification dispersion and profile control function diagram. (**a**): Initial state; (**b**): Emulsification and dispersion; (**c**): Emulsion profile control. The purple areas represent rock, while the orange areas represent crude oil or crude oil emulsion.

The profile-control agent achieves multi-stage conformance control through the synergistic combination of aqueous-phase viscosity enhancement and emulsion plugging. On one hand, intermolecular hydrophobic associations and electrostatic interactions endow the agent with excellent viscosity-building capabilities under reservoir temperature and salinity conditions. This increased viscosity reduces aqueous phase mobility, improves the oil-water mobility ratio, suppresses water channeling, and promotes a more uniform displacement front. On the other hand, the agent effectively emulsifies crude oil to form large-droplet O/W emulsions. As illustrated in Figure 19, when the emulsion droplet size is commensurate with the pore-throat dimensions, it plugs the corresponding throats, creating physical barriers within preferential flow paths. The combined action of viscosity increase and emulsion blockage elevates flow resistance in these high-permeability channels, redistributing the pressure gradient. This diverts the displacing fluid into previously unswept, smaller pores, mobilizes residual oil, and expands the sweep efficiency, thereby recovering additional oil left behind by water flooding.

A key aspect is the concentration-dependent nature of these mechanisms. The viscosity-enhancing effect is dominant in the near-wellbore region, where the agent concentration is high. As the agent propagates deeper into the reservoir, its concentration decreases due to adsorption and dilution, thereby weakening its contribution to viscosity. However, even at lower concentrations, the agent retains its effective emulsifying capability. Thus, in the reservoir’s deep regions, the “emulsion plugging” mechanism becomes the primary means of flow diversion. This transition from viscosity-dominated to emulsion-plugging-dominated control enables effective multi-stage conformance improvement and extends the treatment’s validity period. For field application, it is recommended to optimize the injection strategy by employing slugs of varying concentrations. This involves injecting a small, high-concentration slug of the profile-control agent first, followed by a large, low-concentration slug. This approach achieves high-strength blockage of the high-permeability channels near the wellbore and enables large-scale emulsification and profile control deep within the reservoir, thereby effectively enhancing water flooding recovery.

## 3. Conclusions

This paper presents the development of a graded polymer profile-control agent suitable for high-temperature, highly heterogeneous heavy oil reservoirs. Performance evaluation and microscopic oil displacement mechanism studies demonstrate that a 3000 mg·L^−1^ solution of this agent exhibits an apparent viscosity of 348 mPa·s at 30 °C and 1221 mPa·s at 70 °C. It possesses a unique “low viscosity at low temperature, high viscosity at high temperature” characteristic, ensuring good injectivity, along with excellent viscosity-increasing capability, salt tolerance, and anti-aging properties.

Its core advantage lies in its “graded control” function: in the near-wellbore high-concentration zone, it primarily improves the water-oil mobility ratio by increasing the aqueous phase viscosity, thereby suppressing viscous fingering; in the deep reservoir low-concentration zone, it emulsifies heavy oil to form large-sized emulsion droplets, generating a plugging effect that diverts fluid flow. This process enlarges the sweep volume and extends the effective treatment period. At equivalent apparent viscosity, it increases displacement pressure by 3.5–5 times compared to HPAM flooding.

Due to its exceptional properties, such as “temperature-sensitive characteristics” and the “graded control” mechanism, the graded polymer profile control agent developed in this study can significantly reduce application costs in oilfields and effectively broaden the application limits for reservoirs. It holds significant importance for the economic stable production of inefficient heavy oil reservoirs, including those with strong edge/bottom water and highly heterogeneous reservoirs after multiple cycles of steam stimulation.

## 4. Materials and Methods

### 4.1. Materials

**Figure 20:**
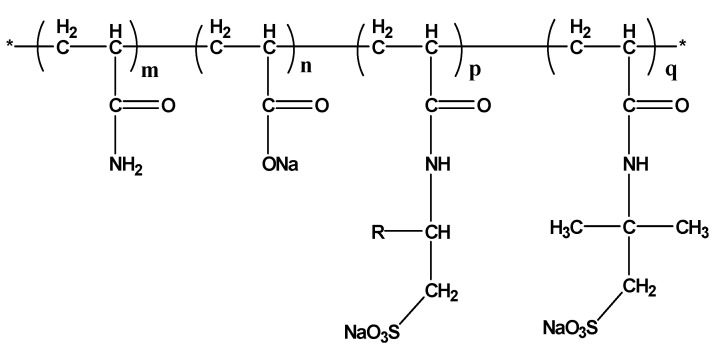
Molecular structure of profile-control agent. * Represents a repeating unit.

The profile-control agent was synthesized in the laboratory via the polymerization of acrylamide, 2-acrylamido-2-methylpropanesulfonic acid (AMPS), and 2-acrylamidohexadecanesulfonic acid (AMC16S). Its relative molecular weight, as determined by Size Exclusion Chromatography with Multi-Angle Light Scattering (SEC-MALS), is 19 million, and it has a hydrolysis degree of 40%. The molecular structure is shown in Figure 20. 2-Acrylamidohexadecanesulfonic acid (AMC16S) was purchased from Shandong Weifang Songchuan New Materials Co., Ltd. (Shouguang, China). Partially hydrolyzed polyacrylamide (HPAM), with a relative molecular weight of 25 million and a hydrolysis degree of 30%, was purchased from Shandong Baomo Bio-Chemical Co., Ltd. (Dongying, China).

The heavy oil sample used in the experiment was Gudao crude oil. The viscosity of the degassed crude oil at 50 °C was 4356 mPa·s, and the contents of the SARA four components were as follows: w(asphaltene) = 2.61 wt%, w(resin) = 20.55 wt%, w(aromatic hydrocarbon) = 23.63 wt%, and w(saturate) = 38.56 wt%.

The water sample used in the experiment was formation water from Gudao Oilfield, with a salinity of 4857.9 mg·L^−1^. The mass concentrations of Na^+^ + K^+^, Ca^2+^, Mg^2+^, Ba^2+^, HCO_3_^−^, SO_4_^2−^, CO_3_^2−^ and Cl^−^ were 1693, 68.7, 55.2, 2.578, 350.1, 31.8, 30.1 and 2626.42 mg·L^−1^, respectively.

The sand-packed models all utilized artificial cemented cores with dimensions of 30 cm × 4.5 cm × 4.5 cm.

### 4.2. Experimental Methods

#### 4.2.1. Critical Association Concentration

The critical association concentration (CAC) was determined using the pyrene fluorescence probe method. A pyrene acetone solution was added to aqueous solutions of the profile-control agent with concentrations ranging from 250 to 1370 mg·L^−1^, resulting in a final pyrene concentration of 1.9 × 10^−6^ mol/L. The emission spectra from 350 to 550 nm were recorded using a fluorescence spectrophotometer at an excitation wavelength of 335 nm, and the intensity ratio of the first peak to the third peak (I_1_/I_3_) was calculated.

#### 4.2.2. Apparent Viscosity

The profile-control agent solution was prepared using a mechanical stirrer at an agitation rate of 300 r/min. After stirring for 2 h, the solution was left to stand for another 2 h. The apparent viscosity of the aqueous agent solution was measured at specified temperatures using an Anton Paar MCR 302 rheometer (The manufacturer, Anton Paar GmbH, is headquartered in Graz, Austria.). Each measurement was performed in triplicate, and the average value was taken. Unless otherwise specified, the apparent viscosity was measured under experimental conditions referenced from Section 7.5 of the enterprise standard [33] with a fixed shear rate of 7.34 s^−1^.

For testing aging resistance, the agent solution was first degassed under a vacuum of −0.1 MPa for 1 h, purged with high-purity nitrogen, and then placed in a 65 °C oven. The apparent viscosity of the solution was measured after different aging periods.

#### 4.2.3. Microscopic Morphology

The microscopic morphology of the aqueous solution of the profile-control agent was observed using scanning electron microscopy (SEM). The sample was rapidly frozen to a vitreous state via high-pressure freezing to inhibit ice crystal formation. It was then fractured under vacuum at −100 °C to expose a fresh cross-section, and surface ice was removed by sublimation. Finally, after conductive coating, the sample was placed on the instrument’s cold stage at −160 °C for in situ observation of the hydrated structure under high vacuum. The concentration of the profile-control agent solution used in the experiment was 3000 mg·L^−1^.

#### 4.2.4. Static Adsorption Capacity

The absorbance of profile control agent solutions at concentrations ranging from 100 to 800 mg·L^−1^ was determined using the starch-chromium iodide colorimetric method, establishing a standard curve for the relationship between the agent’s mass concentration and absorbance. The agent solution and quartz sand were added to a 100 mL reagent bottle at a solid-to-liquid ratio of 1:5 (mass ratio). After purging with nitrogen to remove oxygen, the bottle was sealed and mixed thoroughly, then placed in a thermostatic water bath shaker and oscillated at 50 °C for 48 h. Upon completion of adsorption, the polymer solution was centrifuged at 3500 r/min for 30 min for separation. The supernatant was extracted from the centrifuge tube, diluted to within the 100–800 mg·L^−1^ range, and its concentration was measured. The equilibrium mass concentration was obtained by multiplying this value by the dilution factor.

The static adsorption capacity was calculated according to Formula (1):

(1) $$\Gamma \;=\;\frac{(\rho_{0\;}-\;\rho_{e})V}{G}$$

- Γ is the static adsorption capacity, expressed in milligrams of polymer adsorbed per gram of quartz sand (mg/g);
- *ρ_0_* is the initial mass concentration of the polymer (mg/L);
- *ρₑ* is the equilibrium mass concentration of the polymer after adsorption (mg/L);
- *V* is the volume of the polymer solution (mL);
- *G* is the mass of the quartz sand particles (g).

#### 4.2.5. Emulsion Droplet Size

At 50 °C, an aqueous solution of the profile-control agent was mixed with Gudao crude oil at an oil-to-water ratio of 7:3. A stable O/W emulsion was obtained by mechanical stirring at a rate of 300 r/min for 30 min. A droplet of the emulsion was placed on a glass slide, covered with a coverslip, and observed under an optical microscope. The objective lens magnification was adjusted, and after focusing, images were captured using software. The average droplet size and particle size distribution curve were obtained through image processing with relevant software.

#### 4.2.6. Core Flooding Experiment

Before the experiment, N_2_ was used to check the air tightness of the system, and the steady-state method was used to measure the gas permeability (Experimental methods refer to appendix standard references [34]). During the experiment, the oil sample was injected at the rate of 0.05 mL/min under the constant temperature of 65 °C for 32 h. At 65 °C, 1 PV formation water was injected at a flow rate of 0.2 mL/min, then inject 3.5 PV of polymer solution, and finally conduct 4PV water drive. During the experiment, the produced liquid was collected every 0.5 PV, and the injection volume, produced liquid volume and system pressure were recorded simultaneously. The concentration of profile-control agent solution used in the experiment is 1500 mg/L, and the salinity of brine used for preparing the solution is 6000 mg/L, 30,000 mg/L and 50,000 mg/L, respectively. The concentration of HPAM solution used in the experiment was 2400 mg/L, and the salinity of brine used to prepare the solution was 6000 mg/L. The core permeability is 1080–1310 × 10^−3^ μm^2^.

#### 4.2.7. CT Scanning Experiment

Two artificial cores with different permeabilities were selected. Prior to the experiment, gas permeability was measured using the steady-state method. A dual-core parallel flooding experiment was conducted, followed by CT scanning of the two square core models at the conclusion of different displacement stages. The CT scanning system primarily consists of an X-ray source, a sample holder, an X-ray detector, and a workstation for data storage, processing, and display. The experimental procedure was as follows: First, the cores were saturated with the oil phase (Gudao crude oil). Water flooding was then performed until the water cut reached 90%. Next, 0.5 PV of the profile-control agent was injected, followed by an additional 1 PV of subsequent water flooding. CT scanning was conducted at four specific time points: after oil saturation, after water flooding, after agent flooding, and after subsequent water flooding. High-permeability and low-permeability core CT images for each stage were reconstructed by processing the original signals. The experiment was conducted at 65 °C with a displacement rate of 0.2 mL/min, using a profile-control agent concentration of 1500 mg·L^−1^. The core dimensions were 30 cm × 4.5 cm × 4.5 cm, with a permeability contrast of 3. The low-permeability layer had a permeability of 1250 × 10^−3^ μm^2^, and the high-permeability layer had a permeability of 3750 × 10^−3^ μm^2^.

#### 4.2.8. Microfluidic Displacement Experiment

The experimental chip was first fabricated, and the displacement experiment was subsequently conducted using a high-temperature, high-pressure microfluidic setup. A representative cast thin section from the Gudao Oilfield with high clarity was selected as the chip for the microfluidic displacement experiment. The cast thin section underwent processes including tracing extraction, binarization, and noise reduction. The microfluidic displacement chip was fabricated via wet etching, with dimensions of 4.0 mm × 2.7 mm. The high-temperature, high-pressure microfluidic experimental system primarily consists of an injection system, a confining pressure system, and an image data monitoring and acquisition system. The injection system delivers fluids into the microchannel chip, while the back-pressure system controls the pressure at the outlet. During the experiment, the core was first saturated with the oil phase (Gudao crude oil), followed by water flooding with 1 PV. Then, 0.5 PV of the profile-control agent was injected, followed by an additional 1 PV of subsequent water flooding. The experiment was conducted at 65 °C, with profile-control agent concentrations of 1500 mg·L^−1^ and 3000 mg·L^−1^, respectively. A microscope was used to monitor and record real-time images within the chip, and image processing software was employed for quantitative characterization of the experimental images.

## Figures and Tables

**Table 1:** Apparent viscosity of profile-control agent under different aging time conditions.

Aging Time, d	Profile-Control Agent		HPAM	
	Viscosity, mPa∙s	Viscosity Retention Rate, %	Viscosity, mPa∙s	Viscosity Retention Rate, %
0	1125	100	52	100
7	1097	97.35	39	75
30	1101	97.9	28	53.8
60	1078	95.8	15	28.8
90	1073	95.4	10	19.2

**Table 2:** Dykstra-Parsons coefficients of oil saturation at different development stages.

Stage	High-Permeability Layer Dykstra-Parsons Coefficient	Low-Permeability Layer Dykstra-Parsons Coefficient
Initial saturated oil	0.1	0.2
Water flooding	0.22	0.36
Agent flooding	0.75	0.57
Subsequent water flooding	0.38	0.41

**Table 3:** Statistical table of crude oil emulsification and dispersion.

Chemical Flooding	Average Droplet Size/μm	Number of Particles	Relative Content of Lotion/%
Profile-control agent drive	51.233	172	28.79
HPAM drive	85.981	64	7.43

## Data Availability

The original contributions presented in this study are included in the article. Further inquiries can be directed to the corresponding author.

## Abbreviations

The following abbreviations are used in this manuscript:

CT	Computed Tomography
O/W	Oil-in-Water
CAC	Critical association concentration
PV	Pore volume
HPAM	Partially Hydrolyzed Polyacrylamide
Vdp	Dykstra-Parsons coefficient
